# Attainment of reproductive competence, phase transition, and quantification of juvenility in mutant genetic screens

**DOI:** 10.3389/fpls.2014.00032

**Published:** 2014-02-17

**Authors:** Ianis G. Matsoukas

**Affiliations:** ^1^Engineering, Sports and Sciences Academic Group, The University of BoltonBolton, UK; ^2^Institute for Renewable Energy and Environmental Technologies, The University of BoltonBolton, UK

**Keywords:** *Arabidopsis thaliana*, florigenic and antiflorigenic signaling, heteroblasty and attainment of reproductive competence, juvenile-to-adult phase transition, reciprocal transfer experiments

## Introduction to juvenility

**Figure 1:**
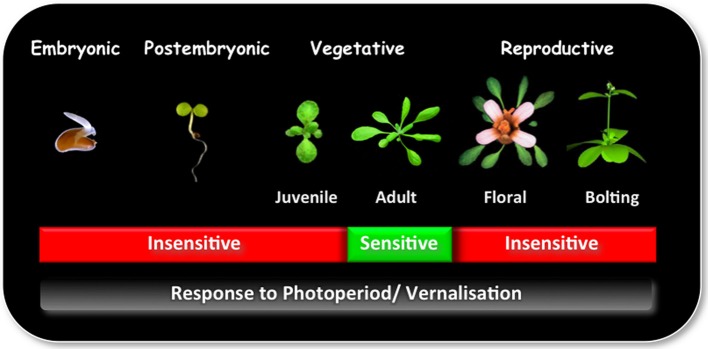
**The ability to sense and respond to photoperiod and/or vernalization varies in different phases of development.** Plants undergo a series of qualitative transitions during their life-cycle in response to environmental and endogenous factors. One of the most distinguishable is the transition from a vegetative to reproductive phase of development, also known as the transition to flowering. This stage is preceded by the juvenile-to-adult phase transition within the vegetative phase. During the juvenile phase plants are incompetent to initiate reproductive development and are effectively insensitive to photoperiod and/ or vernalization. With the change to adult phase, plants attain competence to respond to floral inducers, which is required for the transition to the reproductive phase. Photoperiod is perceived in the leaf, whereas vernalization at the shoot apical meristem.

Plant development between seedling emergence and flowering is characterized by a series of successive qualitative phases: (1) a post embryonic photoperiod-insensitive phase, during which plants are insensitive to photoperiod; (2) a photoperiod-sensitive inductive phase, in which plants require a number of short day (SD) or long day (LD) inductive cycles, depending on their age for rapid flowering, and (3) a photoperiod-insensitive post-inductive phase, in which plant development is no longer influenced by photoperiod (Figure 1; Matsoukas et al., 2013).

The early phase of development during which the plants cannot be induced to flower and are effectively insensitive to environmental influences of photoperiod and/or vernalization has been called the juvenile phase (Thomas and Vince-Prue, 1997). This period differs from plant to plant from a period of a few days, for small herbaceous annual plant species, through to periods that may last longer than 20 years, as is evident for many tree species. From a physio-ecological perspective, by having a juvenile phase, plant species avoid the low seed yields that would occur if they were to flower precociously while still small and with limited photosynthetic capacity (Thomas and Vince-Prue, 1997).

## The importance of the juvenile phase studies

Studies on the juvenile-to-adult phase transition have significant scientific and economic implications. Juvenility has long attracted interest as an aspect of the fundamental topic of aging and also has practical implications, especially in the growth and development of those species in which it is striking and prolonged (Matsoukas et al., 2012). From a commercial perspective, understanding the factors that affect the timing and duration of the juvenile phase length is critical for scheduling in commercial horticulture and arable crops. In addition, the long juvenile phase length of some species is one of several features limiting efficient breeding programs. For example, the efficiency of trait selection and genetic improvement in breeding programs is inversely related to the period of the breeding cycle. Thus, the exploitation of genotypes with short juvenile phase is very important. On the other hand, in many countries fast-growing tree species are being increasingly used for pulp and bioenergy production. In such cases, it may be equally important to explore molecular methods to prevent flowering and prolong juvenility (Matsoukas et al., 2012). Therefore, improving our knowledge of the ways, by which abiotic conditions and genetic factors influence juvenility and floral induction could help with crop scheduling, decrease time to flowering, and reduce waste with resulting benefits for the environment through lower inputs and energy required per unit of marketable product (Matsoukas et al., 2012, 2013).

## Molecular genetics of the juvenile-to-adult phase transition

Molecular genetic analyses have provided insights into mechanisms that regulate the juvenile-to-adult and vegetative-to-reproductive phase transitions in several plant model systems (reviewed in Jansson and Douglas, 2007; Albani and Coupland, 2010; Huijser and Schmid, 2011; Andres and Coupland, 2012; Bolouri Moghaddam and Den Ende, 2013). MicroRNA156 (miR156), an ambient temperature-responsive miR (Lee et al., 2010) and strong floral inhibitor, is one of the central regulators of the juvenile-to-adult and vegetative-to-reproductive phase transitions in several species (Wu and Poethig, 2006; Chuck et al., 2007, 2011; Wang et al., 2011). Functional analysis of the *hasty1* (*hst1*) mutant of *Arabidopsis thaliana* revealed the function of the contrasting transcriptional pattern of the phloem-transmitted miR156 (Lee et al., 2010) and miR172 (Lauter et al., 2005; Martin et al., 2009; Varkonyi-Gasic et al., 2010) in regulation of phase transitions (Wu and Poethig, 2006; Chuck et al., 2007; Jung et al., 2007; Mathieu et al., 2009). It has been shown that the juvenile-to-adult phase transition is accompanied by a decrease in miR156/miR157 abundance and a concomitant increase in abundance of miR172, as well as the SQUAMOSA PROMOTER-BINDING PROTEIN-LIKE (SPL) transcription factors (TFs; Shikata et al., 2009; Wang et al., 2009; Jung et al., 2011; Shikata et al., 2012). Expression of miR172 in leaves activates *FLOWERING LOCUS T* (*FT*; Aukerman and Sakai, 2003; Jung et al., 2007), the final output of the photoperiodic pathway (Corbesier et al., 2007), through repression of AP2-like transcripts *SCHLAFMÜTZE* (*SMZ*), *SCHNARCHZAPFEN* (*SNZ*) and *TARGET OF EAT 1–3* (*TOE1–3*; Jung et al., 2007; Mathieu et al., 2009), whereas the increase in *SPL*s at the shoot apical meristem (SAM), leads to the transcription of floral meristem identity (FMI) genes (Wang et al., 2009; Yamaguchi et al., 2009). The FMI genes trigger the expression of floral organ identity genes (Causier et al., 2010), which function in a combinatorial fashion to specify the distinct floral organ identities.

The juvenile-to-adult phase transition is genetically regulated, although, as with most genetic traits, there are interactions with abiotic factors (Telfer and Poethig, 1998; Mohamed et al., 2010; Bergonzi et al., 2013). *Arabidopsis* genotypes impaired in sugar signaling, starch anabolism and catabolism, and floral repressor mutants show altered juvenile phase lengths compared to their respective wild types (Matsoukas et al., 2013). In addition, examination of diurnal metabolite changes in starch deficient and starch excess mutants indicates that their altered juvenile phase length may be due to lack of starch turnover, which influences carbohydrate availability (Matsoukas et al., 2013). Interestingly, miR156a and miR156c, the major sources of miR156 in *Arabidopsis*, are significantly down regulated by sugars (Yang et al., 2013; Yu et al., 2013). Furthermore, it has been shown that trehalose-6-phosphate (Tre6P) acts as a local signal that links sugar availability to the juvenile-to-adult and vegetative-to-reproductive phase transitions (Wahl et al., 2013). *Arabidopsis* plants impaired in Tre6P signaling pathway are late flowering. This late flowering phenotype was found to be due to reduced expression levels of *FT*, the elevated levels of miR156, and reduced levels of at least three miR156-regulated transcripts, *SPL3*, *SPL4*, and *SPL5* (Wahl et al., 2013).

## Biochemical influence

A number of biochemical changes have been proposed to mark the juvenile-to-adult phase transition in different plant species. For example, differences in peroxidase and esterase isozymes (Brand and Lineberger, 1992) and in protein phosphorylation (Huang et al., 1992). Furthermore, while various hormones have been shown to affect the juvenile-to-adult phase transition, their responses sometime differ. The hormones auxin (De Zeeuw and Leopold, 1955), abscisic acid (Rogler and Hackett, 1975), cytokinin (Mullins et al., 1979) and ethylene (Beyer and Morgan, 1971) have been demonstrated to be involved in the juvenile-to-adult phase transition. In addition, gibberellic acid (GA) has promotional and repressive effects depending on plant species (Wilson et al., 1992; Chien and Sussex, 1996; Telfer et al., 1997; Telfer and Poethig, 1998). In *Arabidopsis*, GA mutations that affect GA biosynthesis (*ga1-3, ga4-1*, and *ga5-1*) and GA sensitivity (*spindly4*) lengthen and shorten the vegetative phase transition, respectively (Telfer et al., 1997). However, it is unclear whether alterations in various hormones levels directly control the juvenile-to-adult phase transition. The action of hormones could be indirect, for instance, by controlling partitioning or mobilization of photosynthates, and/or interacting with other hormones (Domagalska et al., 2010) and sugar signals (Zhou et al., 1998; Moore et al., 2003).

## Morphological, histological, and physiological markers

In some species, the juvenile-to-adult phase transition has also been associated with several morphological, histological, and physiological traits. For instance, leaves may change in shape, size, phyllotaxy, and thickness. Other features associated with the developmental stage may relate to pigmentation, rooting ability, growth habit, orientation of vascular bundles, cold and disease resistance, and the physiological status of the plant (Poethig, 1990, 2003; Brunner and Nilsson, 2004; Itoh et al., 2005). However, these features are not totally reliable, since they are usually affected by different factors such as water availability, temperature, photoperiod, light quality and irradiance. In addition, the morphological, histological, and physiological changes are often less distinct in herbaceous than in woody species, and in many cases no clear association exists (Jones, 1999; Brunner and Nilsson, 2004).

In *Arabidopsis* the appearance of trichomes marks the juvenile-to-adult phase transition (Telfer et al., 1997). Leaves of plants in their juvenile phase of growth produce trichomes only on the adaxial surface, whereas leaves of plants in their adult phase produce trichomes on both the adaxial and abaxial surfaces. However, mutations in *Arabidopsis* affecting trichome development can alter trichome distribution in ways that are not phase specific (Telfer et al., 1997). This complicates the use of trichome distribution as a phase marker in mutant genetic screens.

The term “vegetative phase transition” is currently being used to characterize both heteroblasty and attainment of reproductive competence, since the two developmental events occur during the vegetative growth that precedes the transition to the reproductive phase (Poethig, 1990, 2009). However, by assessing morphological characteristics, several plant species undergo the vegetative-to-reproductive phase transition while still displaying juvenile traits, and others in which floral induction does not occur, even if adult traits are appeared and the plants are treated with photo- and/or thermo-inductive conditions (Brunner and Nilsson, 2004; Poethig, 2010). This could indicate that estimation of the length of the juvenile phase based on morphological traits does not necessarily provide a reliable indication of when juvenility ends. Therefore, the use of the terms “juvenile vegetative phase” and “adult vegetative phase” in defining both the heteroblastic transition as well as the state of floral competence may lead to perplexity.

## Competence to respond to floral inductive signals: a reliable determinant that can be used to quantify the length of the juvenile phase

The juvenile-to-adult phase transition is affected by several abiotic conditions (Matsoukas et al., 2013) and so chronological time (or the number of dormancy cycles) does not necessarily provide a reliable indication of when juvenility ends. Floral competence is the most robust determinant that can be used to distinguish between the juvenile and adult vegetative phases of plant development. However, non-flowering plants are not necessarily in their juvenile phase of development; they might be floral competent but have not been exposed to photo- and/or thermo-inductive conditions for flowering.

A simple method of quantifying the length of the juvenile phase accurately and reproducibly is to conduct reciprocal transfer experiments (Mozley and Thomas, 1995; Matsoukas et al., 2013). This approach involves transferring plants at regular intervals between conditions that are inductive and non-inductive for flowering, for example between LDs and SDs (or between different levels of temperature; response to vernalization), and assess leaf number and flowering time responses (Adams et al., 2001, 2003). This approach enables the analysis of reciprocal transfer experiments data in terms of the following parameters: (1) the photoperiod-insensitive juvenile phase; (2) the photoperiod-sensitive floral inductive phases in both SDs and LDs; and (3) the photoperiod-insensitive post inductive phase (Adams et al., 2003). Plants transferred from non- or less inductive conditions to inductive conditions before the end of juvenility will exhibit similar flowering times (and for terminal flowering plant species, have the same leaf numbers), as those grown constantly in the inductive conditions (Adams et al., 2003). On the other hand, floral induction will be delayed in plants that remain under non-inductive conditions after juvenility has ended. Experimental data sets obtained by the reciprocal transfer approach can be analyzed by fitting models such as those described by Adams et al. (2001, 2003). The reciprocal transfer approach has the advantage that it can be used on small seedlings, where grafting techniques are impractical, and in species where genetic analyses are not possible as little is known about the genetic regulation of the juvenile-to-adult phase transition.

## Concluding remarks

The juvenile-to-adult and vegetative-to-reproductive phase transitions regulated by multiple pathways, which show different responses to external and internal stimuli. Much of the evidence for the various factors involved in the juvenile-to-adult phase transition can be subject to multiple interpretations. However, it can be proposed that the prolonged juvenile-to-adult and vegetative-to-reproductive phase transitions might be due to a plethora of antiflorigenic signals, which affect the transcription levels of *FT* and *SPL*s. Therefore, juvenility can be defined as the period during which the abundance of antiflorigenic signals such as miR156/miR157 is sufficiently high to suppress the expression of *FT* and *SPL* genes.

Determination of the length of the juvenile phase is a complex issue. The estimation of juvenility based on morphological, physiological, histological and biochemical markers does not necessarily provide a reliable indication of when juvenility ends. The exploitation of a single and simple experimental system to obtain accurate and reproducible estimates regarding the length of juvenility in different plant species is of crucial importance. Reproductive competence is a robust determinant that can be used to distinguish between plants that are juvenile or adult. This can be determined by conducting reciprocal transfer experiments. The simplicity of this approach enables its application in diverse plant species with comparative ease, including on young seedlings, and in genotypes where the practice of grafting is unfeasible.
